# Evaluation of the Available Variant Calling Tools for Oxford Nanopore Sequencing in Breast Cancer

**DOI:** 10.3390/genes13091583

**Published:** 2022-09-03

**Authors:** Asmaa A. Helal, Bishoy T. Saad, Mina T. Saad, Gamal S. Mosaad, Khaled M. Aboshanab

**Affiliations:** 1 Department of Bioinformatics, HITS Solutions Co., Cairo 11765, Egypt; 2 Department of Microbiology and Immunology, Faculty of Pharmacy, Ain Shams University, Organization of African Unity St., Cairo 11566, Egypt

**Keywords:** nanopore, variant detection, human-SNP-wf, Clair3, Clair, NanoCaller, Longshot, Medaka

## Abstract

The goal of biomarker testing, in the field of personalized medicine, is to guide treatments to achieve the best possible results for each patient. The accurate and reliable identification of everyone’s genome variants is essential for the success of clinical genomics, employing third-generation sequencing. Different variant calling techniques have been used and recommended by both Oxford Nanopore Technologies (ONT) and Nanopore communities. A thorough examination of the variant callers might give critical guidance for third-generation sequencing-based clinical genomics. In this study, two reference genome sample datasets (NA12878) and (NA24385) and the set of high-confidence variant calls provided by the Genome in a Bottle (GIAB) were used to allow the evaluation of the performance of six variant calling tools, including Human-SNP-wf, Clair3, Clair, NanoCaller, Longshot, and Medaka, as an integral step in the in-house variant detection workflow. Out of the six variant callers understudy, Clair3 and Human-SNP-wf that has Clair3 incorporated into it achieved the highest performance rates in comparison to the other variant callers. Evaluation of the results for the tool was expressed in terms of Precision, Recall, and F1-score using Hap.py tools for the comparison. In conclusion, our findings give important insights for identifying accurate variants from third-generation sequencing of personal genomes using different variant detection tools available for long-read sequencing.

## 1. Introduction

Over time, the field of genetic testing for many cancer biomarkers, such as breast cancer driver genes BRCA1 and BRCA2, improved, starting from single gene sequencing on sanger sequencing technology, followed by multigene panels, which were created as a result of developments in next-generation sequencing technology (NGS), allowing for a broader genetic assessment, a faster testing method, and better throughput, without being cost prohibitive but constrained by the generation of short reads [1,2]. MinION, the first long-read Nanopore-based sequencer, was released by Oxford Nanopore Technologies (ONT), overcoming the primary limitations of short-read sequence creation [3] by introducing long-read sequencing technology that was adapted by both ONT and Pacific Biosciences (PacBio) [4]. These technologies proved that new long-read, single-molecule sequencing technologies could reliably be able to identify small variants, indel, and structural variants (SVs), with significant improvements in both sensitivity and specificity [3,5].

In human genomes, single-nucleotide polymorphisms (SNPs) and short insertions and/or deletions (indel) are two forms of genetic variants [6,7]. They contribute to genetic diversity and have the ability to affect phenotypic differences, such as human disease susceptibility. Detecting SNPs and indel is challenging in studying genomic variants and functions using new generations of high-throughput sequencing data [5]. Many different variant (SNP/indel) callers were introduced by the Nanopore community and recommended by ONTs for accurate variant detection based on data from long-read sequencing. Some variant callers implemented variant calling methods using deep learning, such as “Clair” [8], the successor of “Clairvoyant” [9]. “Longshot “ [10] calls SNPs on long-read data using a Pair-Hidden Markov Model (pair-HMM) for a small local window surrounding candidate sites. Medaka [11], an SNP/indel caller based on deep learning on long-read data, was recently launched by ONTs [11]. Medaka predicts SNPs from unphased long reads before phasing them. For each set of phased reads, Medaka ends up making SNP and indel calling. Nanocaller [12] is a deep convolutional neural network that incorporates a long-range haplotype structure to improve variant detection on long-read sequencing data. “Clair3” [13] combines the greatest characteristics of two key method categories: pile-up calling, which handles most variant candidates fast, and full alignment, which tackles complicated candidates with precision and recall in an account. Accordingly, in this article, the development of a workflow for detecting disease-causing variants, starting from the sample to the variant call format (VCF) with annotated variants, was proposed where different variant calling tools were tested on reference genome samples to evaluate the output of each tool against “Truth” set of variants. The proposed pipeline for targeted sequencing of the data generated from long-read sequencing technology, where the two genes BRCA1 and BRCA2, which are recurrently mutated in breast cancer, were analyzed as an example of this workflow and an examination of its performance was described for future testing and implementation.

## 2. Materials and Methods

### 2.1. Targeted Sequencing Data Analysis Pipeline

The target amplicons’ reads were aligned to reference sequences based on the public human genome build GRCh38/UCSC hg38 using Minimap2 Aligner trained on long reads generated by ONT-MinIon sequencer (https://github.com/lh3/minimap2 (accessed on 8 August 2022) [14]. After Minimap2 finishes the alignment, it generates a SAM file that is converted afterward to BAM format using Samtools (https://github.com/samtools/ (accessed on 8 August 2022) [15]. The resultant BAM file was sorted and indexed using Samtools to be ready for variant calling. The minimum sequencing depth value was found to never be below 50 X using Bedtools “coverage” (https://github.com/ryanlayer/bedtool (accessed on 8 August 2022) [16]. Afterward, the PCR duplicate removal was performed on the reads that have identical external coordinates, retaining only the reads with the highest mapping quality using Samtools rmdup (with s option) that removes the single-end reads from the sorted and indexed Bam file (https://github.com/samtools/ (accessed on 8 August 2022)) [15]. Regarding the variant calling step, six variant callers were tested in parallel on the MinIon sequencing data: (1) Medaka (https://github.com/nanoporetech/medaka (accessed on 8 August 2022)) [11], (2) epi2me-labs/wf-human-snp (https://github.com/epi2me-labs/wf-human-snp (accessed on 8 August 2022)) [17], (3) Clair3 https://github.com/HKU-BAL/Clair3 (accessed on 8 August 2022) [13], (4) Clair (https://github.com/HKU-BAL/Clair (accessed on 8 August 2022)) [8], (5) Longshot (https://github.com/pjedge/longshot (accessed on 8 August 2022)) [10], (6) Nanocaller (https://github.com/WGLab/NanoCaller (accessed on 8 August 2022)) [12]. A custom-made BED file was created to target the region of the BRCA1 and BRCA2 genes for the variant callers to call only variants in our target regions. The ‘SNV’ (single-nucleotide variant) and ‘INDEL’ (insertion–deletion) files were filtered by removal of non “PASS” variants and with Quality “QUAL” below 20. The filtered VCF of variants was then annotated using the Genetic variant annotation and functional effect prediction toolbox SnpEff (https://pcingola.github.io/SnpEff/ (accessed on 8 August 2022)) [18], which predicts the effects of the resultant variants on genes and amino acid changes. The ClinVar (https://www.ncbi.nlm.nih.gov/clinvar/ (accessed on 8 August 2022)) [19] database was used to check for the clinical significance of the annotated variants. The database is strongly linked to the databases dbSNP and dbVar, which keep track of the site of variations in human assembly. ClinVar is based on the phenotypic descriptions kept in MedGen (http://www.ncbi.nlm.nih.gov/medgen (accessed on 8 August 2022)) [20] as well. The SNV and INDEL variants that were clinically significant are reported and stored in the in-house database (Table 1).

### 2.2. Classification of the Pathogenicity of Variants

The information deposited in the ClinVar database and the recommendations of the American College of Medical Genetics and Genomics (ACMG) were used to classify the detected mutations [21,22]. The results of the BRCA1/2 gene variant detection were classified as wild type (no harmful variants), variant of unknown significance (VUS), pathogenic variants (PV), and likely pathogenic variants (LPV); not all the benign variants were reported [23].

### 2.3. Validation Data Set

To ensure the pipeline’s usefulness and readiness, two long-read datasets based on publicly accessible human reference samples HG001 (NA12878) (https://www.ncbi.nlm.nih.gov/popset/?term=NA12878 (accessed on 8 August 2022)) and HG002 (NA24385) (https://www.ncbi.nlm.nih.gov/genome/?term=NA24385 (accessed on 8 August 2022) were provided by the ONT-open-data registry that is provided to support: (1) exploration of the properties of Nanopore sequence data; (2) performance evaluation and replication; (3) tool and method development. These are two of the most used reference samples. The Fastq files provided along with the bam files for this sample were used as input to test the validity of different tools’ output (https://registry.opendata.aws/ont-open-data/ (accessed on 8 August 2022)) using the benchmarking tool (https://github.com/Illumina/hap.py (accessed on 8 August 2022)) [24].

## 3. Results

### 3.1. Data Analysis Workflow Outcome

Data analysis workflow for the HG001 and HG002 reference genomes started with the read sequence aligner Minimap2, which aligns DNA sequences against the GRCh38 human reference genome with a SAM file as an output. Samtools “View” was used to convert the SAM file to a BAM file, followed by Samtools “Sort” and “Index” to generate a sorted and indexed BAM file ready for variant calling. As a part of the workflow pipeline, a step of PCR duplicate removal from the aligned reads of the two reference samples was included, to avoid overestimation of the coverage and overestimated variants resulting from PCR duplication with Samtools “rmdup”. The mean coverage was calculated by bedtools “coverage”. For the sample HG001, the mean coverage for the reads before PCR-duplicate removal was 32.62 X and 36.89 X for BRCA1 and BRCA2, respectively, while after removal of PCR duplicates, the mean coverage for BRCA1 and BRCA2 was found to be the same. The mean coverage for HG002, before the PCR-duplicate removal, was 53.85 X for BRCA1 and 70.06 X for BRCA2. After removing the duplicates, the mean coverage of BRCA1 and BRCA2 was found to be the same, which suggested that the published reference samples previously underwent the step of PCR-duplicate removal or it was sequenced as a whole-genome sequencing sample, which is more logical (Table 2).

### 3.2. Primary Filtering Outcomes

The BAM files were ready for the next step, which was the variant calling step. Six tools were used to call variants in the BRCA genes in HG001 and HG002; some of these tools were recommended by ONT and some by the ONT community for variant calling, such as Medaka, Clair, Nanocaller, Longshot, Clair3, and wf-human-snp workflow, which is the workflow provided by ONT employing Clair3 with pre-adjusted parameters for accurate variant calling. All of the generated output VCFs were filtered, including the variants with “PASS” and QUAL > 20 as a threshold for the comparison of the output of the tools. Long-read sequencing data aligned to a reference genome are taken as an input along with a BED file designed to target the two genes’ coordination, which restricts the variants called in the target location into different variant callers, which output a VCF file with predicted SNPs and indel. The output after the primary filtering for the three samples is described in Table 3 and Table 4.

### 3.3. Comparison of the Variant Caller’s Performance

For a comparison of the variant caller’s performance, the traditional binary classification performance assessment paradigm of simply determining true and false “positives” and “negatives” lends itself well to evaluating the performance of variant callers [6]. By comparing the results to the truth sets for the NA24385 sample or NA12878 sample using the Hap.py tool that enumerates the variants between a “truth” VCF file containing the truth set of variants and a “query” VCF file, which contains the set of output variants of the variant caller along with a BED file that restricts the comparison to variants in the specified target location to determine the reliability of the variant calling conducted. The hap.py tool outputs a summary with the true positive “TP”, false positive “FP”, false negative “FN”, Precision, Recall, or sensitivity, and finally, F1-score, which is an indication and a representation of both precision and recall. The data generated from the comparison tool “Happy” were summarized to include important metrics, such as Recall, Precision, F1-score, and the time taken by the tool to call the variants in both genes (Table 5 and Table 6). With respect to the time taken for the tools to perform the variant calling on only the coordination of BRCA1 and BRCA2 genes for each sample, Nanocaller proved to be faster in this aspect where the time taken for Nanocaller was the lowest and Clair was proved to take the longest time in two samples HG001 and HG002 (Table 5 and Table 6). 

## 4. Discussion

Evaluation of BRCA1/2 molecular status has become the standard of care in the treatment of individuals with breast cancer. Precision medicine has made significant progress against this type of cancer, which accounts for one-third of all new female cancers every year. Female breast cancer is the sixth biggest cause of mortality worldwide, with an estimate of 685,000 deaths in 2020 [25]. One example is the development and clinical application of PARP-inhibitor (PARPi); Poly (adenosine diphosphate-ribose) polymerase inhibitors (PARPi) are a key arrow in the oncologist’s quiver among new therapeutics [26,27]. Indeed, PARPi has been found to enhance the clinical outcomes of breast cancer patients with BRCA1/2 germline or somatic mutations, which have been found to improve survival and quality of life [28,29,30,31,32]. As a result, current worldwide guidelines strongly advise BRCA1/2 testing in all patients. Rapid and dependable genetic screening for BRCA1/2 germline or somatic mutations has become critical in identifying individuals who would most likely benefit from these treatments [3,33,34].

The technology used in BRCA 1/2 gene testing held an important impact on getting the full picture of the two genes. Traditional Sanger sequencing is expensive and takes a long turn-around time (TAT). Next-generation sequencing (NGS) is a game-changing high-throughput nucleotide sequencing approach that produces rapid, cheap, and accurate genomic data. NGS developed the clinical methodology for genetic examination across various fields of medicine [34]. NGS can massively sequence millions of DNA reads, allowing for accurate characterization of the “status” of multiple genes; in this context, NGS-targeted gene sequencing enables the detection of driver mutations, which are responsible for progression and relapse and might be employed as predictive or prognostic biomarkers in breast cancer [33,34]. When compared to Sanger sequencing, NGS can offer doctors comparable genetic information at a cheaper cost and shorter time to results [2,34], yet the NGS limitations are the small read size and the difficulty in analyzing large alterations as structural variants. Many studies employed the NGS as a technology in the detection of BRCA1/2 gene variants in various ethnic groups to implement the detection of gene variants using NGS in the routine line of diagnostics and may allow doctors to make more prompt and informed decisions about surgery or neo-adjuvant chemotherapy in breast cancer patients [35,36,37,38,39,40,41,42].

However, the use of NGS technologies in clinical diagnostics necessitates a large initial investment in the sequencer, which is a barrier for local research institutions in underdeveloped nations, as well as small research institutes and hospitals. MinION, the first commercially available sequencer based on Nanopore technology, might be a viable alternative [43,44]. MinION has previously been utilized effectively to identify mutations in TP53 and ABL1 genes in CLL and CML patients [45,46,47,48], respectively. Furthermore, the cheap cost, ease of use, and length of the reads make MinION a perfect instrument for targeted gene sequencing; the long read can enable researchers to detect and phase genetic variants, as well as thoroughly define new isoforms and fusion transcripts, using Nanopore technology. Nanopore technology sheds new light on health and disease, ranging from cancer to immunology and neurology [48].

In the current study, the main focus was on the data analysis of data generated using Nanopore technology, as there are many proposed tools by the Nanopore community, a hub for all the Nanopore technology users (https://community.nanoporetech.com/ (accessed on 8 August 2022)) for every step along the way in data analysis. The in-house targeted gene sequencing workflow was divided into two parts: (1) design a data analysis pipeline for SNV/INDEL/SV detection and how to validate this pipeline and (2) design an in-house primer panel for BRCA1/2 genes as a prototype for future implementation. The pipeline design started with a set of tools designed and trained on long-read data generated from the MinIon ONT sequencer; the reference samples used as the input data for validation of this workflow are the publicly published “NA12878” (HG001) reference sample [49] and “NA24385” (HG002) dataset that contain whole-genome sequencing of well-known human cell lines, sequenced using Nanopore technology [50]. Each, therefore, serves as a helpful benchmark sample. The HG002 cell line was used as a “seen” sample in the current (PrecisionFDA Truth Challenge V2) competition [51]. The method of validating the performance of workflows and especially the variant callers is called “Benchmarking”, where a reference sample is used either as DNA to be sequenced and undergo the workflow or using the data for this reference sample from the public repository in-silico for a data analysis step, a method that was recommended by Global Alliance for Genomics and Health (GA4GH) [52].

The pipeline went as follows: (1) mapping for the reads stored in the fastq file that outputs the reads into a SAM file format using “Minimap2” mapper for long reads against reference sequences based on the GRCh38/UCSC hg38 public human genome build, (2) sorting and indexing using Samtools as a versatile tool as it was heavily used in many pipelines proposed by other studies, used to convert a SAM file to BAM file, sort and index the BAM output, (3) removing the PCR duplicates even though the reference data samples used to validate this workflow were whole-genome sequencing, not including a PCR step but were included in the workflow as this workflow will be used on targeted gene sequencing data, (4) calculating the mean coverage of the targeted genes using Bedtools, (5) variant calling step, which is the main event in the workflow and the focus of our study; there are many variant callers both recommended by ONT and the Nanopore community, so the output variants were filtered based on “PASS” and QUAL > 20 as a threshold for the comparison of the tools output, (6) annotating the variants using SnpEff as an annotation tool, and (7) checking the clinical significance of the annotated variants using ClinVar clinical database.

The focus of the current study was to evaluate this workflow as well as compare the performance of the commonly used software pipelines for variant calling, which is another key element in variant discovery. The comparison is based on how well the tool calls the “True” variants when compared to the benchmarking VCF file; the tools analyzed in this study are Medaka, Clair, Nanocaller, Longshot, Clair3, and ONT’s wf-human-snp workflow for variant calling, which employs Clair3 with pre-adjusted parameters for the accurate calling of variants.

Recent studies attempted to enhance variant calling by using phasing information from long-read sequencing data. Longshot calls SNPs on long-read data using a pair-hidden Markov Model (pair-HMM) for a small local window surrounding candidate sites and then improves genotyping of identified SNPs using Hap-CUT2 [53] based on the most probable pair of haplotypes given the present variant genotypes, but on the other hand, is incapable of detecting indel. Medaka was provided by ONT, an SNP/indel caller that uses deep learning on long-read data. Medaka predicts SNPs from unphased long readings before using WhatsHap [54] to phase the data Medaka eventually makes SNP and indel calls for each phased read group. Clair, the successor of Clairvoyante, is a tool for detecting germline minor variants quickly and accurately using single-molecule sequencing data. Clair outperforms several competing systems for ONT data, including Clairvoyante, Longshot, and Medaka, in terms of precision, recall, and speed. As a deep learning approach, Nanocaller detects SNPs using long-range haplotype information, then phases long reads with identified SNPs and calls indels using local realignment.

Two key designs differ greatly in terms of performance and speed either employing pileup or full alignment as the input of the decision-making neural network. Clair and Nanocaller are pileup-based calling networks that aggregate read alignments into features and counts before sending them into a variant calling network. PEPPER-Margin-DeepVariant5 (PEPPER) [55] is fully alignment based. The DeepVariant variant calling network input is retained with spatial information in the full alignment method and is tens of times greater in size than the pileup method. Medaka is consensus based, using pileup input to generate a diploid consensus in the first iteration and two haploid consensuses in the second. Variants are formed by identifying and combining differences between the reference and consensus. To fill the void, Clair3 was created, which combines the best of both designs. It is as quick as pileup-based callers and performs just as well as full alignment callers. First, the pileup calling network goes through all the variant candidates that met a coverage and alternative allele frequency criterion. The high-quality pileup calls are then used to phase the alignments and generate the final output. Then, for each low-quality pileup call for full-alignment calling, the alignments phased by WhatsHap are utilized to create full-alignment input that is 23-times greater in size than the pileup input. Finally, as the final output, the full-alignment calls are combined with the high-quality pileup calls.

For performance validation of the pipeline along with the variant callers, the process started with the genome in a bottle (GIAB) reference samples HG001 and the Ashkenazi son sample HG002 ONT reads that were used as an input for mapping with Minimap2, sorting and indexing with Samtools, calculating the mean coverage within the BRCA1/2 gene bed file with coordination. for the variant calling step, the default parameters were used for all the variant callers to ensure uniformity in the output variants. The benchmarking variant VCF “Truth set” used was the GIAB v.4.2.1 for each reference genome sample to compare the output of different variant callers. The hap.py [24] tool was used for benchmarking, which is a reference implementation of the GA4GH recommendations for variant caller benchmarking with the “vcfeval” engine for comparison; it generated metrics as “False positive”, “False negative”, “True positive”, “Precision”, “Recall”, and “F1 score”. It was found that three metrics are the most important for variant caller performance evaluations, which are “Precision”, “Recall”, and, most importantly, “F1 score”, which is the mean of precision and recall and is commonly used to test the performance of the callers [56,57,58].

Based on the metrics obtained in our results, it is suggested that Clair3 as a stand-alone or incorporated into a workflow as Human-SNP-wf by ONT, was found to be outperforming other variant callers concerning performance. The Clair3 method’s efficiency is based on its ability to effectively distinguish between true and false calls during pileup calling, allowing only essential candidates to be transferred to the considerably more computationally costly full alignment calling. Following that comes Nanocaller, which performed in a better way than the rest of the variant callers, Longshot, Clair, and Medaka, respectively, agree with the findings of another study. Even though Clair is supposed to outperform Longshot, it was found to have lower F1 scores in both reference samples and that may be because Clair was outdated and was succeeded by Clair3 in May 2021 (https://github.com/HKU-BAL/Clair (accessed on 8 August 2022)). Although Medaka was, up until the release of Clair3, the recommended variant caller for SNP calling using the “medaka_variant” argument, which was formerly implemented inside the medaka package, it has been exceeded in accuracy and computing performance by alternative approaches and is, thus, deprecated and it is advised to utilize Clair3 either directly or through the Oxford Nanopore Technologies offered Nextflow implementation (Human-SNP-wf) (https://github.com/nanoporetech/medaka (accessed on 8 August 2022)) and that may explain the low performance. It was intentional not to test Nanopolish [59], which is also capable of variant calling on ONT data since it requires fast5 raw signals file as input, which are not publicly accessible for HG002, so it was excluded from the variant callers’ comparison.

Targeted gene panels are one of the most frequent ways of enriching the genomic areas to be sequenced and they are widely utilized in NGS technology. Using Nanopore technology, we were able to enrich all the gene areas of interest without being limited by the read length. MinION real-time sequencing allows reads to be evaluated as they are produced, considerably speeding up analysis and allowing for the modification of experimental conditions as needed. Another benefit of MinION over second-generation sequencers is its mobility and ease of use for library preparation and sequencing, as well as its low cost. There are currently many custom/academic or commercial BRCA1/2 target panels that have been established in recent years because of investigations on the use and impact of NGS in breast/ovarian cancer [56,60,61,62], the majority of which are based on the amplicon sequencing technique. There are currently many commercial short-read amplicon-based BRCA gene panels available that detect SNV and/or copy number variation. Nonetheless, efforts to create a complete gene panel useful for BRCA prognosis and medication impact prediction are ongoing. The design of a primer panel targeting different oncology biomarkers will be incorporated into our future plan for trial on different cancer sample types.

## 5. Conclusions

In this study, six variant calling tools, including Human-SNP-wf, Clair3, Clair, NanoCaller, Longshot, and Medaka, were evaluated regarding their performance and accuracy in the detection of genetic variants. The tested genetic variants were single-nucleotide polymorphisms (SNPs) and short insertions and/or deletions (indel) of BRCA1 and BRCA2 genes, where two reference genome sample datasets (NA12878) and (NA24385) were used. The set of high-confidence variant calls provided by Genome in a Bottle (GIAB) was used to allow for the evaluation of the performance of six variant calling tools. The obtained results provide important insights for identifying accurate variants from third-generation sequencing of personal genomes using different variant detection tools available for long-read sequencing. The evaluation of the results was expressed in terms of Precision, Recall, and F1-score using Hap.py tools for the comparison. Both Clair3 and Human-SNP-wf tools accomplished the highest performance rates and should be implemented for evaluating the prognosis of breast cancer in humans.

## Figures and Tables

**Table 1:** Summary of the tools used in both SNP and indel detection.

Tool	Version	Function
Guppy	v5.0.16	data processing toolkit that contains Oxford Nanopore’s base-calling algorithms. Guppy is integrated into MinKNOW and is also available as a standalone version.
Minimap2	v2.22	A sequence alignment tool that aligns DNA or mRNA sequences to a vast library of reference sequences.
Samtools	v.1.14	a collection of programs for manipulating alignments in the SAM, BAM, and CRAM formats. It converts between formats, sorts, merges, and indexes data, it can quickly remove PCR duplicates and calculate the mean coverage for a target region
Medaka	v1.4.4	a program that uses Nanopore sequencing data to generate consensus sequences and calling of variants.
Clair	v2.11	a tool that uses single molecule sequencing data to call germline small variants quickly and accurately.
Longshot	v0.4.1	a tool for detecting variants in diploid genomes using long error-prone reads. It takes an aligned BAM/CRAM file as input and outputs a phased VCF file containing variant and haplotype information.
NanoCaller	v2.1.2	a computational method for detecting SNPs/indels in long-read sequencing data that integrates long reads in a deep convolutional neural network and generates predictions for each SNP candidate variant site by considering pileup information from other candidate sites that share reads.
Clair3	v0.1-r11	a long-read germline small variant caller excels in two major method categories: pileup calling, which handles most variant candidates quickly, and full alignment, which tackles complex candidates to maximize precision and recall.
Hap.py	v0.3.15	To compare a VCF with a gold standard dataset vcf
SnpEff	v5.1	Toolbox for genetic variant annotation and functional effect prediction. It describes and estimates the effects of genetic variants on genes and proteins (such as amino acid changes)
Epi2me-labs/wf-human-SNP	v0.3.1	includes a nextflow workflow for calling diploid variants in whole genome data. Clair3 is used in this workflow to identify small variants in long reads.

SAM: Sequence Alignment Map, BAM: Binary Alignment Map, CRAM: Compressed Reference-oriented Alignment Map, VCF: Variant call format

**Table 2:** The coverage difference before removing duplicates and after removing duplicates.

Sample	Before Removing Duplicates		After Removing Duplicates	
	BRCA1	BRCA2	BRCA1	BRCA2
HG001	32.62 X	36.89 X	32.55 X	36.89 X
HG002	53.85 X	70.06 X	53.85 X	70.06 X

**Table 3:** The total no. of the output variants (SNPs, INDELs, and MNPs) of the six variant callers in comparison to both BRCA1 and BRCA2 genes in the HG001.

Tool Name	Total No. of BRCA1 Variants	Total No. of BRCA2 Variants	Total
Clair	482	348	830
Longshot	124	108	232
NanoCaller	121	97	218
Medaka	221	221	442
Clair3	225	172	397
Epi2me-labs/wf-human-SNP	370	285	655

**Table 4:** The total no. of the output variants (SNPs, INDELs, and MNPs) of the six variant callers in comparison to both BRCA1 and BRCA2 genes in the HG002.

Tool Name	Total No. of BRCA1 Variants	Total No. of BRCA2 Variants	Total
Clair	482	372	854
Longshot	124	108	232
NanoCaller	121	97	218
Medaka	111	98	209
Clair3	370	172	542
Epi2me-labs/wf-human-SNP	370	285	655

**Table 5:** Summary for the benchmarking output for HG001 with 6 different variant callers, highlighting the recall, precision, and F1-score.

	HG001 (NA12878)	Recall	Precision	F1 Score	Total Time Taken
1. Human-SNP-wf	BRCA1-SNP	98.04%	95.24%	96.62%	1 h
	BRCA1-INDEL	94.12%	80.00%	86.49%	
	BRCA2-SNP	95.24%	96.15%	95.69%	
	BRCA2-INDEL	94.74%	75.00%	83.72%	
2. Clair3	BRCA1-SNP	99.02%	96.19%	97.58%	1 h 22 min
	BRCA1-INDEL	94.12%	80.00%	86.49%	
	BRCA2-SNP	96.19%	97.12%	96.65%	
	BRCA2-INDEL	94.74%	81.82%	87.80%	
3. Medaka	BRCA1-SNP	92.16%	89.52%	90.82%	1 h 29 min
	BRCA1-INDEL	58.82%	50.00%	54.05%	
	BRCA2-SNP	94.29%	95.19%	94.74%	
	BRCA2-INDEL	57.89%	50.00%	53.66%	
4. Nanocaller	BRCA1-SNP	96.08%	93.33%	94.69%	42 min
	BRCA1-INDEL	76.47%	65.00%	70.27%	
	BRCA2-SNP	95.24%	96.15%	95.69%	
	BRCA2-INDEL	80.00%	54.55%	64.86%	
5. Longshot	BRCA1-SNP	95.10%	92.38%	93.72%	48 min
	BRCA1-INDEL	70.59%	60.00%	64.86%	
	BRCA2-SNP	93.33%	94.23%	93.78%	
	BRCA2-INDEL	68.42%	59.09%	63.41%	
6. Clair	BRCA1-SNP	96.08%	93.33%	94.69%	2 h
	BRCA1-INDEL	64.71%	55.00%	59.46%	
	BRCA2-SNP	93.33%	94.23%	93.78%	
	BRCA2-INDEL	63.16%	54.55%	58.54%	

**Table 6:** Summary for the benchmarking output for HG002 with 6 different variant callers, highlighting the recall, precision, and F1-score.

	HG002 (NA24385)	Recall	Precision	F1-Score	Total Time Taken
1. wf-Human-SNP	BRCA1-SNP	97.20%	99.05%	98.11%	43 min
	BRCA1-INDEL	93.33%	70.00%	80.00%	
	BRCA2-SNP	97.06%	98.02%	97.54%	
	BRCA2-INDEL	95.00%	90.48%	92.68%	
2. Clair3	BRCA1-SNP	96.26%	98.10%	97.17%	1 h 7 min
	BRCA1-INDEL	86.67%	65.00%	74.29%	
	BRCA2-SNP	95.10%	96.04%	95.57%	
	BRCA2-INDEL	85.00%	80.95%	82.93%	
3. Medaka	BRCA1-SNP	91.59%	93.33%	92.45%	39 min
	BRCA1-INDEL	60.00%	45.00%	51.43%	
	BRCA2-SNP	90.20%	91.09%	90.64%	
	BRCA2-INDEL	60.00%	57.14%	58.54%	
4. Nanocaller	BRCA1-SNP	95.33%	97.14%	96.23%	28 min
	BRCA1-INDEL	80.00%	60.00%	68.57%	
	BRCA2-SNP	94.12%	95.05%	94.58%	
	BRCA2-INDEL	85.00%	80.95%	82.93%	
5. Longshot	BRCA1-SNP	94.39%	96.19%	95.28%	38 min
	BRCA1-INDEL	73.33%	55.00%	62.86%	
	BRCA2-SNP	92.16%	93.07%	92.61%	
	BRCA2-INDEL	75.00%	71.43%	73.17%	
6. Clair	BRCA1-SNP	93.46%	95.24%	94.34%	1 h 11 min
	BRCA1-INDEL	66.67%	50.00%	57.14%	
	BRCA2-SNP	91.18%	92.08%	91.63%	
	BRCA2-INDEL	65.00%	61.90%	63.41%	

## Data Availability

In publicly accessible repositories, the Nanopore sequencing data have been deposited. The data can be found here: https://www.ncbi.nlm.nih.gov/sra/PRJNA865100 (accessed on 8 August 2022).

## Abbreviations

ACMG: American College of Medical Genetics and Genomics; BAM, Binary Alignment Map; BED, Browser Extensible Data; BRCA1, Breast cancer gene 1; BRCA2, Breast cancer gene 2; ClinVar, Database that aggregates information about genomic variation and its relationship to human health; CRAM, Compressed Reference-oriented Alignment Map; dbSNP, Database of Single Nucleotide Polymorphisms; dbVar, Database of human genomic structural variation; DSB, Double-strand breaks; GA4GH, Global Alliance for Genomics and Health; GIAB, Genome in a Bottle; LPV, Likely Pathogenic Variant; NGS, Next-generation sequencing; ONT, Oxford Nanopore Technologies; PacBio, Pacific Biosciences; PCR, Polymerase chain reaction; PV, Pathogenic variant; SAM, Sequence Alignment Map; SNP, Single-nucleotide polymorphisms; SNV, Single-nucleotide variant; SV, Structural variants; VCF, Variant call format; VUS, Variant of unknown significance.
